# Development of Ciprofloxacin-Loaded Bilosomes In-Situ Gel for Ocular Delivery: Optimization, In-Vitro Characterization, Ex-Vivo Permeation, and Antimicrobial Study

**DOI:** 10.3390/gels8110687

**Published:** 2022-10-25

**Authors:** Omar Awad Alsaidan, Ameeduzzafar Zafar, Mohd Yasir, Sami I. Alzarea, Mohammed Alqinyah, Mohammad Khalid

**Affiliations:** 1 Department of Pharmaceutics, College of Pharmacy, Jouf University, Sakaka 72341, Saudi Arabia; 2 Department of Pharmacy, College of Health Sciences, Arsi University, Asella 396, Ethiopia; 3 Department of Pharmacology, College of Pharmacy, Jouf University, Sakaka 72341, Saudi Arabia; 4 Department of Pharmacology and Toxicology, College of Pharmacy, King Saud University, Riyadh 11451, Saudi Arabia; 5 Department of Pharmacognosy, College of Pharmacy, Prince Sattam Bin Abdulaziz University, Al-Kharj 11942, Saudi Arabia

**Keywords:** ocular delivery, ciprofloxacin, bilosomes, in-situ gel, HET-CAM, antimicrobial

## Abstract

Conventional eye drops are most commonly employed topically in the eye for the management of bacterial conjunctivitis. Eye drops have a low corneal residence time and 90–95% of the administered dose is eliminated from the eye by blinking and the nasolacrimal drainage system. This problem can be minimized by formulating a mucoadhesive ocular in-situ gel system that undergoes sol-gel transition upon stimulation by temperature, pH, and ions. The goal of this study was to develop ciprofloxacin (CIP) loaded bilosomes (BLO) in-situ gel for the improvement of therapeutic efficacy. The BLO was prepared by the thin-film hydration method and optimized by the Box–Behnken design. Cholesterol (CHO), surfactant (Span 60), and bile salt (sodium deoxycholate/SDC) were used as formulation factors. The vesicle size (nm) and entrapment efficiency (%) were selected as responses (dependent factors). The optimized CIP-BLO (CIP-BLO-opt) formulation displayed a vesicle size of 182.4 ± 9.2 nm, a polydispersity index of 0.274, a zeta potential of −34,461.51 mV, and an entrapment efficiency of 90.14 ± 1.24%. Both x-ray diffraction and differential scanning calorimetry spectra did not exhibit extensive peaks of CIP in CIP-BLO-opt, revealing that CIP is encapsulated in the BLO matrix. The CIP-BLO-opt formulation was successfully incorporated into an in-situ gel system using a gelling agent, i.e., Carbopol 934P and hydroxyl propyl methyl cellulose (HPMC K100 M). CIP-BLO-opt in-situ gel formulation (CIP-BLO-opt-IG3) was evaluated for gelling capacity, clarity, pH, viscosity, in-vitro CIP release, bio-adhesive, ex-vivo permeation, toxicity, and antimicrobial study. The CIP-BLO-opt-IG3 exhibited satisfactory gelling properties with a viscosity of 145.85 ± 9.48 cP in the gelling state. CIP-BLO-opt-IG3 displayed sustained CIP release (83.87 ± 5.24%) with Korsmeyer–Peppas kinetic as a best-fitted model (R2 = 0.9667). CIP-BLO-opt-IG3 exhibited a 1.16-fold than CIP-IG and a 2.08-fold higher permeability than pure CIP. CIP-BLO-opt-IG3 displayed a significantly greater bio-adhesion property (924.52 ± 12.37 dyne/cm^2^) than tear film. Further, CIP-BLO-opt-IG3 does not display any toxicity as confirmed by corneal hydration (76.15%), histology, and the HET-CAM test (zero scores). CIP-BLO-opt-IG3 shows significantly higher (*p* < 0.05) antimicrobial activity against P. aeruginosa and S. aureus than pure CIP. From all these findings, it could be concluded that CIP-BLO-opt-IG3 might be an effective strategy for the increment of corneal residence time and therapeutic activity of CIP.

## 1. Introduction

Over the last two decades, there have been a lot of interest in ophthalmic drug delivery. The pharmaceutical market for ophthalmic preparation is on the rise, with different types of dosage forms in single or combination and medical devices being introduced, and yet ideal drug delivery for ophthalmic preparations is still a big challenge [1]. The eye is a complex and sensitive organ in the body. Enhancement of ocular bioavailability of the drug by topical application of formulation is challenging because of various protection mechanisms (eyelids and tear film) against external hostilities participated to eliminate the drug [2]. In addition, nasolacrimal drainage and blinking reflexes are also responsible for the elimination of drugs from the eye after topical application [3].

Certainly, under normal physiological environments, the lacrimal fluid (7 µL) is changed every five minutes, and the secretion can reach 400 µL/min when irritation persists [1,2]. For this reason, the conventional eye drop is diluted rapidly and disregarded from the ocular surface and conjunctival cul-de-sac. However, the ocular surface is intermittently cleaned by eyelid blinking (20 blinks/min) [3,4], resulting in a short contact time of formulation with the cornea, conjunctiva, and sclera. For these reasons, conventional eye drops exhibit poor ocular bioavailability, and a frequently repeated dose is required to achieve the desired pharmacological activity.

To overcome these limitations of conventional eye drops or other conventional ophthalmic preparations, it is preferable to design a novel formulation that can stay in the eye for an extended period and release the drug to achieve the desired concentration. Different types of formulations have been reported by researchers to improve corneal residence time, such as contact lenses, ointments, collagen shields, and ocular inserts [5]. Of course, all of these delivery systems are capable of increasing the bioavailability and therapeutic efficacy of drugs but have some drawbacks, such as patient noncompliance, difficulty in administration, and bullered vision. Colloidal drug delivery is an advanced carrier system for increasing the therapeutic efficacy of the drug after ocular administration. There are various antibiotics-loaded nano-formulations for topical ophthalmic delivery that have been reported to improve therapeutic efficacy, such as besifloxacin-loaded liposome [6], CIP-loaded liposome [7], besifloxacin-loaded nanostructured lipid carrier [8], CIP- loaded nanoemulsion [9], vancomycin loaded niososme [10], and moxifloxacin loaded bilosomes [11]. Among them, bilosomes (BLO) are a novel and advanced carrier system for the enhancement of the ocular delivery of drugs.

BLO is a bile salt-stabilized bilayer closed vesicle made up of a nonionic surfactant similar to niosomes but containing bile salt [12]. BLO is an ultra-deformable flexible vesicle and improves the oral delivery of bovine serum albumin [13]. BLO enhances the stability and encapsulation efficiency of the therapeutic agent [14]. Further incorporation of nanoformulation into an in-situ gel system using gelling polymers further increased corneal contact time and transit time in the cul-de-sac as well as reduced the elimination of drugs. In-situ gel formulation existed in sol form under normal conditions but transformed into gel after topical administration into the eye by stimulation of ions, pH, and temperature [15].

CIP is a fluoroquinolone broad-spectrum antibiotic (acts against both Gram-positive and Gram-negative bacteria) and is widely used for the treatment of ophthalmic topical infections [16]. It prevents the bacterial DNA from uncoiling and duplicating by inhibiting DNA gyrase and topoisomerase IV, resulting in cell death [17]. It also passes the cell wall and cytoplasmic membrane of the Gram-positive and outer membrane of Gram-negative bacteria [18]. CIP is a pale yellow, crystalline powder with 35 mg/mL aqueous solubility [19]. The log P of CIP is 0.28, and the molecular weight is 331.34 g/mol [20]. However, current CIP preparation has two chief drawbacks, i.e., low ocular residence time and low patient acceptability because of high dosing frequency [21].

In the present study, a mixture of carbopol 934 P and hydroxyl propyl methylcellulose (HPMC) was used for the preparation of the in-situ gel. Carbopol is a polyacrylic acid (PAA) based polymer and exhibited sol to gel state in an aqueous solution at a pH raised above the pH of 5.5. It is a weak acidic polymer, and under alkaline pH it ionizes. Thus, at a high pH, the PAA swells because of the electrostatic repulsion of anion groups, releasing the incorporated drug into the medium. It exhibited mucoadhesive properties by interacting with mucin through electrostatic interaction, hydrogen bonding, hydrophobic interaction, and interdiffusion [22]. The acidic nature of Carbopol may produce eye irritation; so, we added HPMC as a viscosity-enhancing agent to decrease the Carbopol concentration [23,24]. HPMC is a semisynthetic, inert, nonionic, nontoxic, and viscoelastic polymer [2].

Extensive research into CIP-loaded bilosome in-situ gel-based formulation has not been reported yet. The goal of this study was to design a CIP-loaded BLO in-situ gel system for the improvement of ocular residence time. The CIP-BLO was developed by the thin film hydration technique and optimized by Box–Behnken design (BBD). Finally, the optimized CIP-BLO was incorporated into an in-situ gel system using macromolecular gelling polymers, i.e., Carbopol 934 P and HPMC 100 K. Finally, the formulation was evaluated for various physicochemical parameters, in-vitro release, ex-vivo permeability, toxicity, and antibacterial activity.

## 2. Result and Discussions

### 2.1. Preliminary Screening

BLO was successfully prepared by the thin film hydration method. In the present study, the screening of different formulation factors was done. Based on preliminary results, the selection of cholesterol, surfactant, bile salt, sonication time, and hydration time was done. The preliminary study results revealed that a very low concentration of cholesterol was not able to form the vesicle. Cholesterol provides rigidity to the vesicle wall. At very high concentrations, the vesicle size and release were also affected. Similarly, the selection of surfactant was assessed by evaluating the size and encapsulation efficiency. At very low and high concentrations of surfactant, the encapsulation was found to be significantly low. At low concentrations, the surfactant was not able to cover all particles but at high concentrations, the drug leaches out from the prepared bilosomes due to the less availability of space for the accommodation of the drug. Similar to surfactants, bile salt also significantly affects the vesicle size and encapsulation efficiency due to its surface-active property. From the preliminary study, the lower and higher concentration of span 60, cholesterol, and bile salt was selected for further optimization by BBD. The effect of sonication and hydration time was also evaluated during the preliminary study but these factors were not chosen for further optimization as their effect was less prominent as compared to selected factors.

### 2.2. Optimization

**Figure 1:**
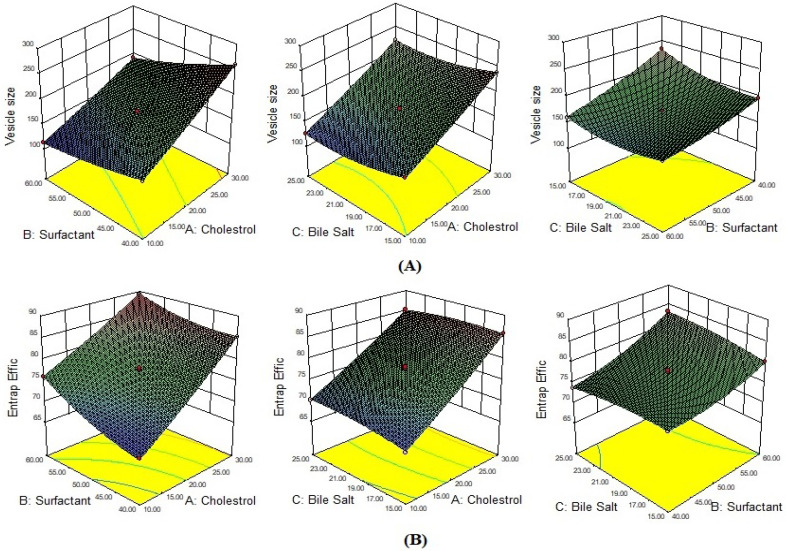
Effect of independent variables *viz* CHO, Span 60, and SDC on (**A**) Vesicle size, and (**B**) Entrapment efficiency.

The BBD was employed for the optimization of CIP-BLO. The composition of all CIP-BLO formulations and their values of responses are given in Table 1. The value of each response was fitted into linear, second-order, and quadratic models. The quadratic model was the best-fitted for each response because it has a maximum R^2^ value (Table S1). Table 2 represented the ANOVA results of each response of the quadratic model and revealed that it is considerably fitted with the highest R^2^, acceptable precision, and low coefficient of variance. Further, the lack of fit is non-significant (*p* > 0.05), indicating less difference between actual and predicted values (Table 2). Figure 1A,B depicted a 3D response graph of all responses that demonstrated the effect of the formulation factors on vesicle size and EE either alone or in combination. The actual and predicted, residual and predicted, and residual and run values of responses are shown in supplementary Figure S1A–F.

#### 2.2.1. Effect of Formulation Variables on VS

The quadratic model-based polynomial equation for VS is presented below:

VS = + 171.04 + 47.04A − 26.52B − 9.69C − 8.23AB + 1.52AC + 8.72BC + 2.28A^2^ + 4.94B^2^ + 11.57C^2^ (1)

Here, the effects of three independent variables, viz., CHO (A), surfactant (B), and SDC (C), were observed on the VS. The equation displayed that model terms, viz., A, B, C, AB, AC, BC, A^2^, B^2^, and C^2^ have a substantial (*p* < 0.05) effect on the VS (Equation (1)). F-value was 1937.80, revealing that the quadratic model was significantly fitted with a non-significant lack of fit (F = 2.17, *p* = 0.2347). The details of the statistical model summary and ANOVA are depicted in Table S1 and Table 2, respectively. A 3D graph (Figure 1A) displayed the influence of variables on VS.

The VS of CIP-BLO batches is in the range of 113.30 ± 7.9 nm (CIP-BLO3) to 259.69 ± 18.1 nm (CIP-BLO2) (Table 1). It was observed that at a fixed concentration of span 60 and SDC, the CHO had a participatory influence on VS, i.e., by increasing the CHO concentration, the VS of BLO increased (batch CIP-BLO1 and CIP-BLO2, CIP-BLO3 and CIP-BLO4). CHO made the compact packing of lipid vesicles, subsequent in an increased aqueous phase within the BLO and an increase in the VS of BLO [25]. The surfactant (Span 60) had the opposite influence on VS. As surfactant concentration increased, the BLO size decreased (batch CIP-BLO1 and CIP-BLO3, CIP-BLO2 and CIP-BLO4) because interfacial tension among CHO and the aqueous phase decreased. The third variable, SDC, had the opposite effect on VS because it reduces the surface tension and the elasticity of BLO (CIP-BLO5 and CIP-BLO7, CIP-BLO6 and CIP-BLO8) [26].

#### 2.2.2. Effect of Formulation Variables on Percent EE

The quadratic model-based multinomial equation for EE is presented below:

EE (%) = + 77.59 + 7.97A + 3.38B + 0.33C − 1.32AB − 0.67AC + 1.23BC + 0.47A^2^ + 1.32B^2^ − 0.69C^2^ (2)

The equation showed that all model terms, viz., A, B, C, AB, AC, BC, A^2^, B^2^, and C^2^ have a substantial (*p* < 0.05) influence on EE (Equation (2)). The F-value is 514.71, revealing that the quadratic model is suggestively fitted. The insignificant lack of fit (F = 1.73, *p* = 0.2984), reveals that the model is well-fitted. The details of the statistical model summary and ANOVA parameters are depicted in Table S1 and Table 2, respectively. A 3D graph (Figure 1B) displayed the influence of all three independent variables on EE.

The EE of CIP-BLO batches is in the range of 67.06 ± 2.7% (CIP-BLO1)–88.06 ± 1.9% (CIP-BLO4) (Table 1). It was found that at a fixed concentration of span 60 and SDC, the CHO exhibited a positive influence on the EE of CPI in BLO, i.e., on increasing the CHO concentration, the EE of CPI in BLO increased (batch CIP-BLO1 and CIP-BLO2, CIP-BLO3 and CIP-BLO4) because CHO may improve the lipophilicity and stiffness of BLO membrane. CHO also enhanced permeability and stability, as well as reduced leakage of the drug from BLO [27,28]. Span 60 had a favorable effect on EE. This may be due to the long alkyl chain of span 60 and the solubility of CIP in the lipiodol phase increased, and EE increased (CIP-BLO1 and CIP-BLO3, CIP-BLO2 and CIP-BLO4). SDC exhibited a concentration-dependent effect on EE [14]. Increasing the SDC concentration increased the EE of CIP in BLO, i.e., 68.14 ± 1.4% (CIP-BLO5) to 70.27 ± 2.3% (CIP-BLO7). This is due to a surface-active characteristic of SDC, which may integrate steeply into the bilayer membrane, disturb the acyl chains of the lipid, increase the membrane elasticity, and increase the EE of CIP in BLO [28]. In batches, CIP-BLO6, CIP-BLO7, CIP-BLO9, and CIP-BLO11, the incremental effect of SDC on EE was relatively low or unaffected. It could be because the SDC solubilization effect is limited due to the dominating effect of other factors [29]. As per the previously reported study, a high concentration of bile salt reduced drug entrapment. This might be due to the fluidizing effect of SDC on the bilayer of BLO and may induce drug escape [29,30].

#### 2.2.3. Optimization of CIP-Loaded BLO Formulation

Based on the vesicle size (191.7 ± 14.7 nm) and EE (88.1 ± 1.9%), the CIP-BLO4 was selected from point prediction. The CIP-BLO4 has 30 mg of CHO, 60 mg of Span 60, and 20 mg of SDC, respectively. A further modification was made to obtain a more valid optimized formulation. After modification, the optimized formulation (CIP-BLO-opt) has 35 mg of CHO, 65 mg of Span-60, and 20 mg of SDC, respectively. The actual value of CIP-BLO-opt has a VS of 182.4 ± 9.3 nm and an EE of 90.1 ± 1.2%, whereas the predicted value of VS is 183.8 nm with an EE of 90.9%. The validity of the model was 99.22% and 99.17% for VS and EE, respectively, revealing much fewer differences between the actual and predicted values of the response.

#### 2.2.4. Characterization of CIP-BLO-Opt Formulation

**Figure 2:**
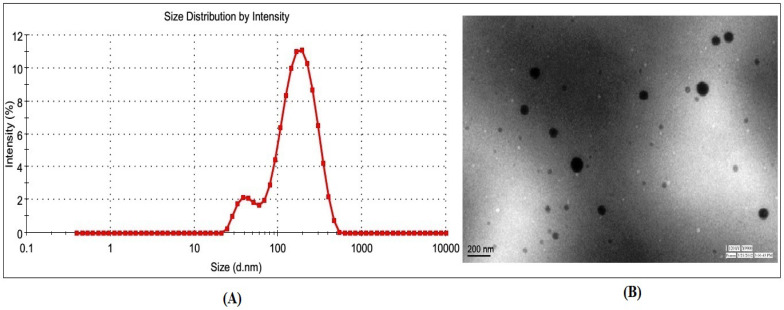
Showing (**A**) Vesicle size graph and (**B**)TEM image of CIP-BLO-opt formulation.

The VS, polydispersity index (PDI), zeta potential, and morphology of CIP-BLO-Opt were evaluated. The CIP-BLO formulations displayed vesicle sizes ranging from 113.30 ± 7.9 nm (CIP-BLO3) to 259.69 ± 18.1 nm (CIP-BLO3) (Table 1). The CIP-BLO-opt formulation displayed a vesicle size of 182.43 ± 9.25 nm (Figure 2A), a PDI of 0.274, and a zeta potential of −34.46 ± 1.51 mV, as well as spherical morphology (Figure 2B), respectively.

#### 2.2.5. Entrapment Efficiency

An adequate amount of drug entrapment in the formulation is accountable for ensuring the desired drug reaches the site of action. A 10 mL sample (equivalent to 6.67 mg of CIP initially as the total initial amount of CIP taken was 10 mg for 15 mL of BLO) of each formulation was taken for the EE study. The EE of all formulations was found to be in the range of 67.06 ± 2.70% (CIP-BLO1) to 88.06 ± 1.90 (CIP-BLO4) (Table 1). The EE of CIP-BLO-opt was found to be 90.1 ± 1.2% (6.00 ± 0.08 mg/ 10 mL of CIP).

#### 2.2.6. DSC Study

Supplementary Figure S2 displays the DSC thermogram of CIP, CHO, SDC, and CIP-BLO-opt. The pure CIP showed a sharp endothermic peak at 311.5 °C, representing its purity and crystallinity (Figure S2A). The characteristic peaks of CHO and SDC were observed at 149.7 °C and 214.9 °C, respectively (Figure S2B,C), assuring their purity. The thermogram of the CIP-BLO-opt (Figure S2D) did not display any CIP peaks, confirming that CIP is encapsulated into the lipid bilayer of BLO. These findings agreed with the previously stated study by Ahmed and co-workers [14]. They developed a BLO of lornoxicam for transdermal delivery using SDC and other formulation components. They demonstrated that lornoxicam transforms into an amorphous state after incorporation into BLO.

#### 2.2.7. Solid-State Characterization by X-ray Diffractometer

**Figure 3:**
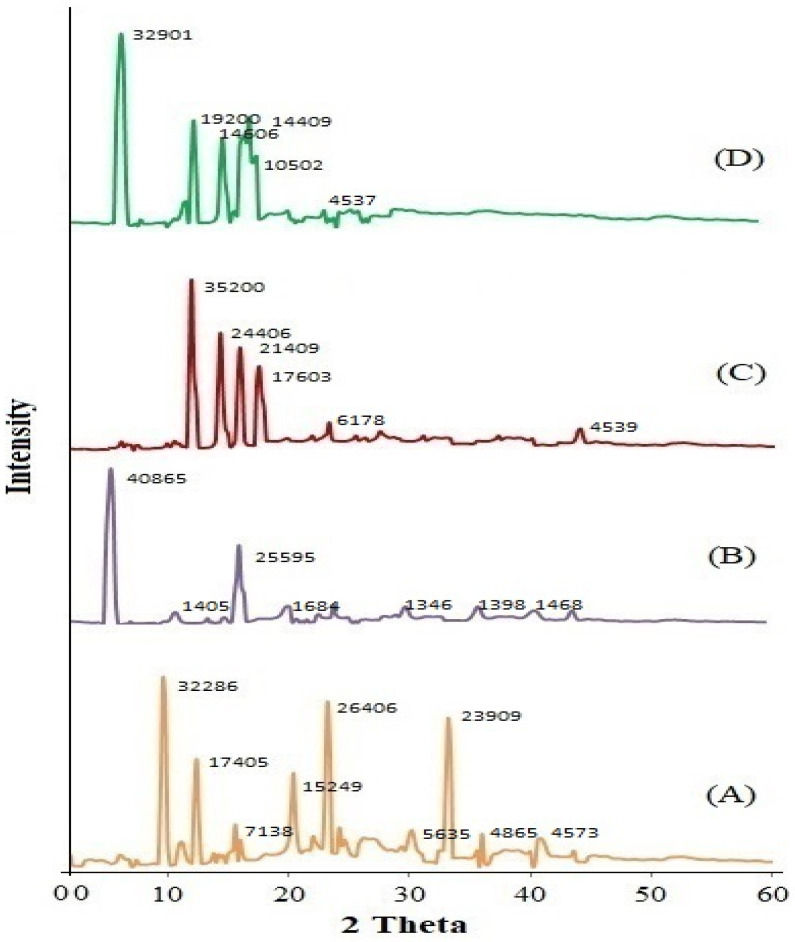
Showing the X-ray diffractogram of (**A**) ciprofloxacin, (**B**) cholesterol, (**C**) sodium deoxycholate, and (**D**) ciprofloxacin-loaded bilosomes.

The diffractogram of CIP exhibited distinctive peaks at 9.6°, 12.4°, 20.4°, 23.6°, 33.2°, etc., confirming its crystalline nature (Figure 3A). The diffractogram of CHO exhibited peaks at 5.4° and 16.2° (Figure 3B), while SDC showed peaks at 11.8°, 14.2°, 15.8°, and 17.4°, respectively (Figure 3C). However, the diffractogram of CIP-BLO-opt (Figure 3D) did not exhibit any distinct peaks of CIP, only some peaks of CHO, and SDC with less intensity, revealing that the CIP was incorporated into the BLO matrix. This result agreed with the findings of Ammar and co-workers [28]. They prepared ondansetron-loaded BLO for transdermal delivery using CHO, Span 60, and SDC and found that loss in crystallinity of ondansetron in the BLO system.

#### 2.2.8. Storage Stability Study

**Figure 4:**
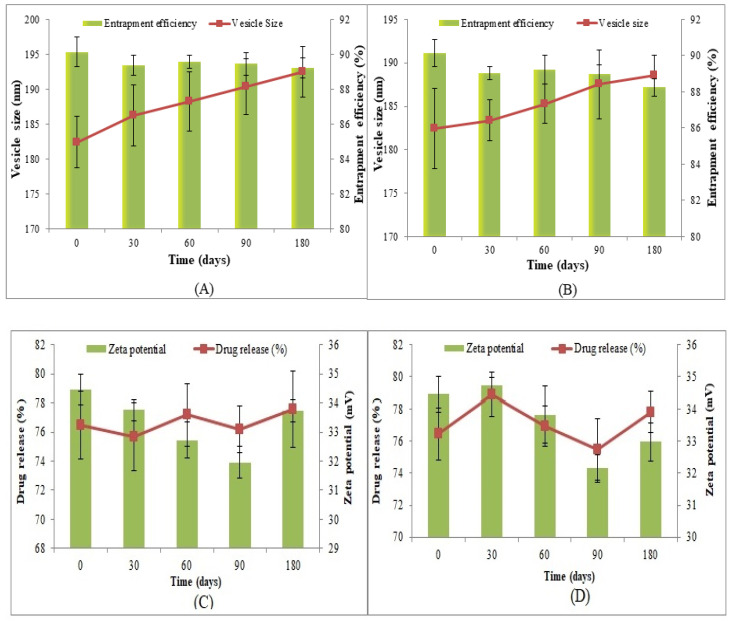
Stability study of CIP-BLO-opt at 4 ±0.5 °C (**A**,**C**) and 25 ± 2 °C/60 ± 5% RH (**B**,**D**). The effect was observed on vesicle size and EE (**A**,**B**) and drug release and zeta potential (**C**,**D**). Data represented as mean ± SD, *n* = 3.

A stability study of CIP-BLO-opt was performed using ICH guidelines. CIP-BLO-opt was found to be clear. There were no significant changes (*p* = 0.065) found in VS and EE at 4 ± 0.5 °C (Figure 4A) and 25 ± 2 °C/60 ± 5% RH (Figure 4B). The reason behind the good stability of CIP-BLO-opt might be due to the high negative charge on SDC (anionic) [30]. Further, the addition of Span 60 also enhanced the stability of the BLO. On the other hand, no significant change was found in the zeta potential of CIP-BLO-opt, i.e., −33.72 ± 1.37 mV (Figure 4C) and −32.98 ± 1.83 mV (Figure 4D), at 4 ± 0.5 °C and 25 ± 2 °C/60 ± 5% RH, respectively, after 180 days of study than zeta potential at zero-day (−34.46 ± 1.51 mV). Initially, CIP-BLO-opt exhibited 34.32 ± 3.57% CIP release in the first 2 h and 76.47 ± 2.83% in 12 h. There was no significant difference found in the % CIP release after storage under specified conditions, i.e., 33.14 ± 2.89% in the first 2 h and 77.54 ± 3.26% in 12 h at 4 ± 0.5 °C. Similarly, at 25 ± 2 °C/60 ± 5% RH, the drug release was found to be 34.98 ± 2.27% (Figure 4D) and 77.83 ± 2.35% in the first 2 h and 12 h, respectively.

#### 2.2.9. Preparation of CIP-BLO-Opt Loaded In-Situ Gel

CIP-BLO-opt was fruitfully converted into an in-situ gel using carbopol-934P (pH-sensitive polymer) and HPMC (viscosity enhancer) polymers.

#### 2.2.10. Characterization of CIP-BLO-Opt-IG

##### Gelling Strength Studies

Gelling strength is a vital parameter of in-situ gel systems for topical ophthalmic delivery. It is defined as the formation of gel immediately in contact with tear fluid and stable for a prolonged time. Gelling strength of the formulation directly influences the ocular retention time, i.e., enhanced corneal residence time. It was determined by adding 100 µL of CIP-BLO-opt-IG formulations to 2 mL of STF in a glass vial and noting the gelation time visually. The results of the gelling strength of all CIP-BLO-opt-IG are depicted in Table 3. CIP-BLO-opt-IG1 formed a gel but dissolved within a minute. On the other hand, CIP-BLO-opt-IG2 formed a gel quickly and was stable for a few hours (>3 h). CIP-BLO-opt-IG3 formed the gel immediately and was stable for >24 h. In the cases of CIP-BLO-opt-IG4 and CIP-BLO-opt-IG5, the gel formed within a few seconds but turned turbid due to high viscosity. Carbopol is a polyacrylic acid (PAA) based polymer with a mucoadhesive property. It exhibits gel-like characteristics as the pH rises (above 5.5). Furthermore, it was noted that on increasing the concentration of polymers, the gelling potential and viscosity of the formulations increased and the system became turbid. Allam and co-workers examined the gelling strength of the betaxolol-loaded niosome in-situ gel system by diluting one drop of the developed formulation with 2 ml of STF [5]. Similarly, Nair and coworkers prepared and determined the gelling strength of moxifloxacin-loaded in-situ gel [15]. In another study, Kesarla and coworkers developed ophthalmic moxifloxacin nanoparticles in-situ gel and evaluated gelling strength by diluting the formulation (50–100 µL) with 2 mL of STF [31].

##### Clarity, pH, and Transmission Measurement

The clarity of all CIP-BLO-opt-IG in the sol state was observed under black and white backgrounds, and the results are depicted in Table 3. CIP-BLO-opt-IG1-CIP-BLO-opt-IG3 did not exhibit any visible particles or precipitation. However, the CIP-BLO-opt-IG4 and CIP-BLO-opt-IG5 formulations are opaque due to the high percentage of gelling agents. The pH of CIP-BLO-opt-IG formulations was near the same in the sol form, i.e., 5.2 ± 0.01. The % transmission of all CIP-BLO-opt-IG was found to be in the range of 81.83 ± 2.48%–98.65 ± 1.92% (Table 3). The percent transmission of the optimized batch (CIP-BLO-opt-IG3) was found to be 98.65 ± 1.92, revealing that the formulation was clear and free from any kind of turbidity and visible particles.

##### Viscosity Determination

The ophthalmic in-situ gel formulation required sufficient viscosity to prevent washout from the ocular region due to blinking of the eyelid (20 blinks/min) [3,4]. The high blinking rates lead to short contact of the formulation with the cornea, conjunctiva, and sclera. The viscosity of the in-situ gel formulation directly influences the gelling potential, gel stability, and release [32]. Table 3 shows the viscosity of all CIP-BLO-opt-IG. The viscosity of formulations in the sol state was in the range of 10.78 ± 1.27 cP (CIP-BLO-opt-IG1) to 128.51 ± 2.03 cP (CIP-BLO-opt-IG5). However, the viscosity of the formulation in the gel state (STF, pH 7.4) was 26.43 ± 3.86 cP (CIP-BLO-opt-IG1) to 431.63 ± 14.23 cP (CIP-BLO-opt-IG5), respectively. CIP-BLO-opt-IG1-CIP-BLO-opt-IG3 had a higher fluidity than CIP-BLO-opt-IG4 but a faster sol-to-gel transition at pH 7.4 at 35 °C [33]. The formulations showed an increased viscosity at pH 5.2 to 7.4 and exhibited pseudoplastic behavior in both environments (non-physiological/during storage and physiological) but had weaker shear-thinning (pseudoplastic) behavior under the same physiological conditions [24]. The in-situ gel system shows shear thinning behavior at ocular shear force (4250–28,500 s^−1^). The HPMC is soluble in water at lower critical solution temperatures (LCST, 50°C) but insoluble at the above LCST and converted into gel [34].

##### Drug Content

The drug content of all CIP-BLO-opt-IG formulations was 94.56 ± 3.26% (CIP-BLO-opt-IG1) to 99.31 ± 1.60% (CIP-BLO-opt-IG4) (Table 3). The drug content of the optimized in-situ gel (CIP-BLO-opt-IG3) is 98.92 ± 2.43% of a total 0.3% *w*/*v* CPI concentration, i.e., 0.29 ± 0.01% *w*/*v* CIP incorporated into the in-situ gel.

#### 2.2.11. Selection of Optimized CIP-BLO-Opt-IG

Based on the characterization parameter results, the CIP-BLO-opt-IG3 was selected as an optimized formulation and the outcomes are shown in Table 3.

#### 2.2.12. In-Vitro Release

**Figure 5:**
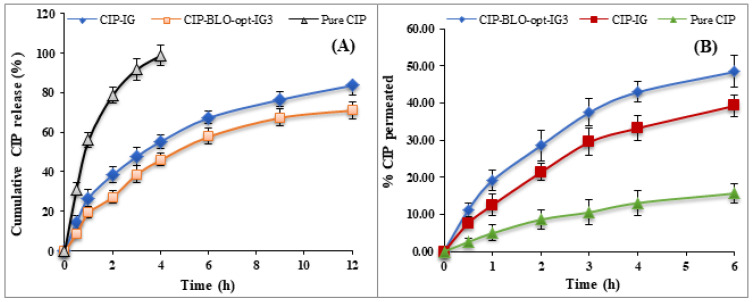
Showing (**A**) In-vitro release profile and, (**B**) ex-vivo permeation study of CIP-BLO-opt-IG3, CIP-IG, and pure CIP. mean ± SD, *n* = 3.

Figure 5A displays the results of in-vitro CIP release from pure CIP, CIP-IG, and CIP-BLO-opt-IG3. The pure CIP (0.3% *w*/*v*) released 78.65 ± 4.62% of CIP than CIP-IG (38.32 ± 4.65%) and CIP-BLO-opt-IG3 (27.28 ± 3.84%) in 2 h. However, nearly 100% (98.63 ± 5.28% in 4 h) of CIP was released from pure CIP. The CIP-IG formulation had a significantly (*p* < 0.05) higher drug release (83.87 ± 5.24%) than CIP-BLO-opt-IG3 (71.16 ± 4.27%) in 12 h. The reason behind the sustained release of CIP from CIP-BLO-opt-IG3 might be the presence of CHO in the bilayer above the SDC, which controls fluidity by constraining CIP mobility and falling the efflux of the incorporated drug [35]. Furthermore, by incorporating CIP-BLO-opt into an in-situ-gel (CIP-BLO-opt-IG3), the CIP release was further slowed and prolonged. This could be due to polymeric hydrogel and higher viscosity (carbopol+ HPMC), which act as an additional barrier to CIP release [19]. The release profile of CIP-BLO-opt-IG3 was put into kinetic release models and the results are represented in Table S2. The R^2^ value was maximum (0.9667) for the Korsmeyer–Peppas model and is selected as the best-fitting model. The exponent n = 0.66, shows the anomalous transport, i.e., CIP release from the CIP-BLO-opt-IG3 via diffusion and relaxation mechanism [36].

#### 2.2.13. Ex-Vivo Trans Corneal Permeation Study

Figure 5B shows the corneal permeation of CIP from the pure CIP, CIP-IG, and CIP-BLO-opt-IG3. The CIP-BLO-opt-IG3 showed a significantly higher permeation across the cornea (48.44 ± 4.33%) than CIP-IG (39.24 ± 2.89%) and pure CIP (15.69 ± 3.69%). The flux of pure CIP, CIP-IG, and CIP-BLO-opt-IG3 was calculated and found to be 112.64 ± 18.80 µg/cm^2^·h, 297.28 ± 21.85 µg/cm^2^·h and 347.76 ± 32.83 µg/cm^2^·h, respectively. The PC of pure CIP, CIP-IG, and CIP-BLO-opt-IG3 was calculated and found to be 6.06 × 10^−4^ ± 1.04 × 10^−4^ cm/min, 1.65 × 10^−3^ ± 1.04 × 10^−4^ cm/min and 1.93 × 10^−3^ ± 1.81 × 10^−4^ cm/min, respectively. Furthermore, the enhancement ratio of CIP-BLO-opt-IG3 was 3.08-fold higher than pure CIP and 1. 16-fold higher than CIP-IG. The CIP-BLO-opt-IG3 showed a significantly (*p* < 0.05) higher flux than pure CIP and CIP-IG due to CHO, surfactant, SDC, nano size of the BLO, and the bio-adhesive nature of the gelling agent. The BLO components may interact with the tear film, may enhance corneal permeability, and extend ocular residence time, preventing CIP loss by tear fluid turnover. The significantly (*p* < 0.05) high permeation of CIP-BLO-opt-IG3 may be due to the foundation of a film over the corneal epithelium release [37]. In addition, the nano size of BLO could be penetrated into the cornea through a receptor-mediated endocytosis mechanism [9].

#### 2.2.14. Bio-Adhesive Study

Bio-adhesiveness of the CIP-BLO-opt-IG3 formulation was determined and found to be 924.52 ± 12.37 dyne/cm^2^ and it was significantly (*p* < 0.05) higher than the shear force of the tear film (150 dyne/cm^2^). The high bio-adhesive power of CIP-BLO-opt-IG3 may increase the corneal contact time and may not be simply washed by the protective mechanism of the eye. Morsi and coworkers reported similar findings in a nano-emulsion incorporated in-situ gel of acetazolamide system using HPMC and Carbopol. They reported that the bio-adhesiveness of the formulation increases with increasing polymer concentration. The mucoadhesive properties of Carbopol and HPMC might be due to the hydrogen bonding between mucin and the carboxylic acid group and the degree of hydration [38].

#### 2.2.15. Corneal Hydration Study

This study represents the tolerance capability of the cornea. The excised goat cornea treated with CIP-BLO-opt-IG3 was employed for assessing this study. For a healthy cornea, the optimum corneal hydration should be 76–80% [39], and a higher value of corneal hydration (>83%) is considered an irritant and is responsible for causing cornea injury [40]. The CIP-BLO-opt-IG3 treated cornea has 76.15 ± 1.46% corneal hydration, and it is under the limit (76–80%), revealing that the cornea was stable throughout the study. It was further assured by histological inspection as well as an ex-vivo ocular tolerance study (HET-CAM).

#### 2.2.16. Histology Study

**Figure 6:**
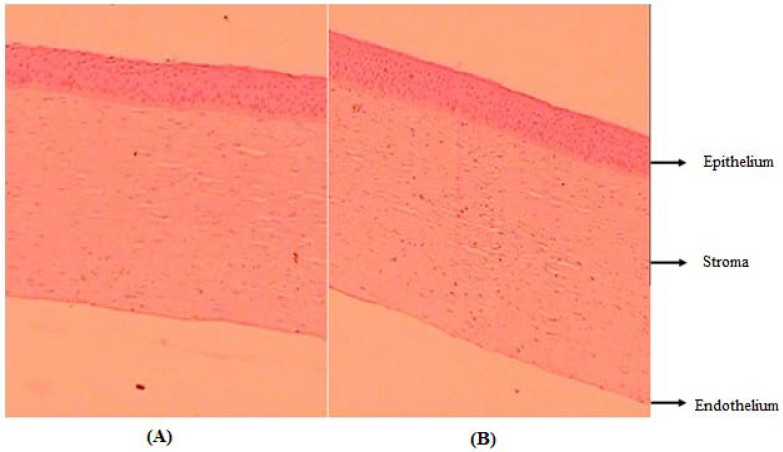
Histology image of treated goat cornea with (**A**) 0.9% *w*/*v* NaCl and (**B**) CIP-BLO-opt-IG3 (10× 10× magnification). No changes were found in the corneal structure after treatment, revealing no irritation.

Figure 6 shows the histological image of an excised goat cornea after treatment with CIP-BLO-opt-IG3 and 0.9% *w*/*v* NaCl (control). There is no damage or irritation observed in the excised goat cornea after treatment with CIP-BLO-opt-IG3 and 0.9% *w*/*v* NaCl (control) (Figure 6A,B). The outcomes of histological evaluation display that the developed formulation or used excipients are safe for ocular administration.

#### 2.2.17. Ex-Vivo Ocular Tolerance Study by HET-CAM

The results of the HET-CAM study are depicted in Table 3S. CIP-BLO-opt-IG3 exhibited no irritation (0.4 scores) without damage to blood capillaries and veins. The 0.9% *w*/*v* NaCl showed a 0.5 score, revealing that no irritation was produced on CAM. However, the irritation score for 0.1M NaOH is 16.93, indicating severe damage to CAM. The result was approved with the previously stated work, i.e., ketoconazole-loaded nanoemulsion in-situ gel [41], and besifloxacin nano-emulsion [42] for ophthalmic administration did not show any damage in CAM.

#### 2.2.18. Biological Compatibility Evaluation

**Figure 7:**
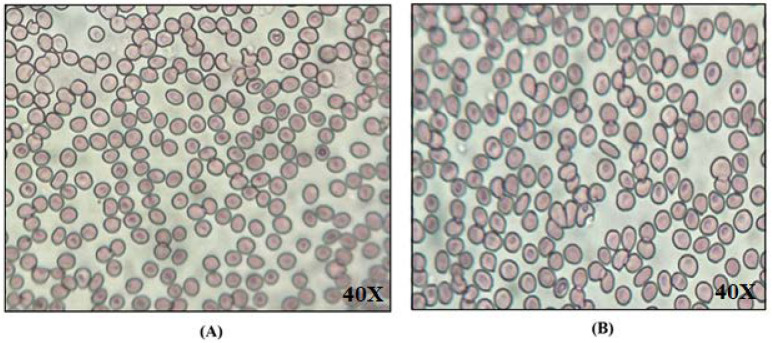
RBCs showing the result of biological compatibility of (**A**) CIP-BLO-opt-IG3 and (**B**) 0.9% *w*/*v* NaCl. The study was performed at 40X.

Figure 7 demonstrates that the RBCs shape (no shrinkage and swelling) was unaffected by the CIP-BLO-opt-IG3 (Figure 7A) as well as the controlled preparation (0.9% *w*/*v* NaCl) (Figure 7B). It revealed that CIP-BLO-opt-IG3 was to be biologically compatible and safe for ocular delivery.

#### 2.2.19. Sterility Study

A sterility test was carried out to see if there was any contamination of anaerobic, aerobic bacteria, and fungi. After 14 days of incubation of the CIP-BLO-opt-IG3 formulation with fluid thioglycolate and soybean casein digest media, it did not show any turbidity or precipitation. It revealed that CIP-BLO-opt-IG3 is sterile.

#### 2.2.20. Antimicrobial Evaluation

**Figure 8:**
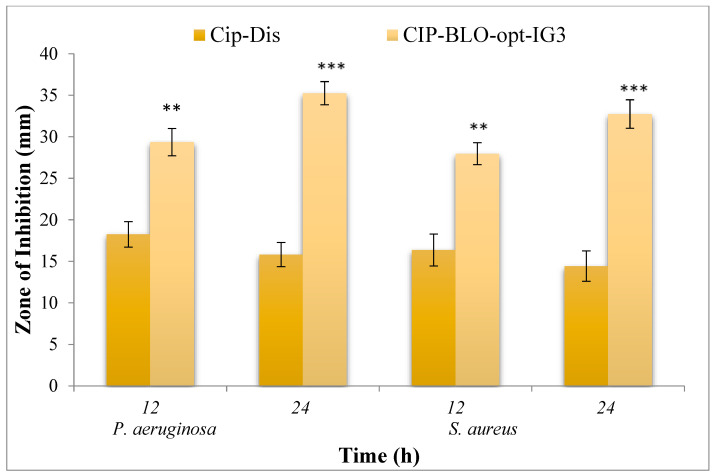
Antimicrobial graph of CIP-BLO-opt-IG3 and CIP dispersion against *P. aeruginosa* and *S. aureus* in the terms of zone of inhibition (ZOI). data are stated as mean ± SD, *n* = 3, **, and *** indicating that CIP-BLO-opt-IG3 is significantly different at 0.05 and 0.01, respectively, from pure CIP.

The cup plate method was applied to assess the comparative antimicrobial effectiveness of CIP-BLO-opt-IG3 and pure CIP against *P. aeruginosa* and *S. aureus*, and the result was depicted in Figure 8. The control sample did not exhibit any antimicrobial activity, while pure CIP displayed a ZOI of 18.25 ± 1.53 and 15.81 ± 1.45 mm in 12 and 24 h, respectively, against *P. aeruginosa.* Similarly, CIP-BLO-opt-IG3 exhibited ZOI 29.36 ± 1.64 mm and 35.25 ± 1.39 mm in 12 and 24 h, respectively, against *P. aeruginosa.* The ZOI was 16.37 ± 1.92 and 14.42 ± 1.83 mm in 12 and 24 h, respectively, for pure CIP and 27.96 ± 1.32 and 32.74 ± 1.71 mm in 12 and 24 h, respectively, for CIP-BLO-opt-IG3 against *S. aureus*. As mentioned previously, the antimicrobial activity of CIP prevents the bacterial DNA from uncoiling and duplicating by inhibiting DNA gyrase in Gram-positive and topoisomerase IV in Gram-negative bacteria [17,18]. The significantly higher (*p* < 0.05) antimicrobial activity of CIP-BLO-opt-IG3 than pure CIP might be due to the nanosize of BLO [29] and BLO ingredients, which is responsible for better diffusion than pure CIP. It is also due to the sustained release of CIP from CIP-BLO-opt-IG3.

## 3. Conclusions

The objective of this research work was achieved by the design of a CIP-BLO in-situ gel with a small VS, PDI, zeta potential, and high EE. The DSC and XRD studies displayed the CIP was encapsulated in the BLO matrix. The optimized BLO formulation was incorporated into the in-situ gel using biocompatible and bio-adhesive polymers, such as Carbopol and HPMC. The CIP-BLO-opt-IG3 quickly transformed into the gel as it came into contact with STF (pH 7.4) and was stable for a prolonged time (>24 h). CIP-BLO-opt-IG3 displayed high viscosity and excellent bio-adhesion. CIP-BLO-opt-IG3 displayed a sustained release of CIP and a suggestively higher flux than pure CIP and CIP-IG. The CIP-BLO-opt-IG3 did not show any signs of toxicity that were confirmed by the corneal hydration study, histology, and HET-CAM test. Furthermore, CIP-BLO-opt-IG3 has a greater antibacterial potential against *P. aeruginosa* and *S. aureus* than pure CIP. Finally, all of the results propose that BLO in-situ gel could be a novel carrier for increasing corneal residency time and therapeutic efficacy of CIP.

## 4. Materials and Methods

### 4.1. Materials

Unicure Pvt. Limited (Noida, India) provided CIP. The cholesterol (CHO), Span 60, and sodium deoxycholate (SDC) were procured from SD Fine Chemicals (Mumbai, India). Acetonitrile, chloroform, methanol, and dialysis bag (MWCO 12kDa) were procured from Sigma Aldrich (Bengaluru, India). Carbopol 934P, Hydroxy propyl methyl cellulose K100 M (HPMC-K100M), Sodium Hydroxide (NaOH), Calcium chloride (CaCl_2_), Sodium Bicarbonate (NaHCO_3_), and Sodium Chloride (NaCl) were procured from SD fine Chem Limited (Mumbai, Maharashtra, India). Fluid thioglycolate and soya bean casein digest media were obtained from HiMedia (Mumbai, India). The other chemicals utilized in this research are analytical grade.

### 4.2. Preparation of CIP-Loaded BLO

A thin-film hydration method was employed for the development of CIP-loaded BLO and developed [43]. The various concentrations of surfactant (Span 60, 40–60 mg), and CHO (10–30 mg) with a fixed dose of CIP (10 mg) dissolved in 10 mL of organic solvent (chloroform, 10 mL) in a round bottom flask. Then, the flask was placed into a rotatory evaporator (BUCHI, Rota vapor R-300, Mumbai, India) to evaporate the organic solvent at 50 °C under reduced pressure. The thin film of the formulation was formed on the surface of a flask and kept in a desiccator overnight for the elimination of moisture. Then, a thin layer was hydrated with an aqueous solution of SDC (15–25 mg in 15 mL) on a rotatory evaporator (1 h at 80 rpm and 40 °C) to obtain the CIP-BLO dispersion. Then, the dispersion was subjected to an ultrasonicator (UP100H, Hielscher Ultrasonics GmbH, Germany) for 5 min in 30 s intervals at 4 °C. The prepared CIP-BLO was stored at 4 °C in collected in borosilicate vials.

### 4.3. Optimization by BBD

Optimization of CIP-BLO was done using 3-factors and 3-levels of BBD, (Stat-Ease, Minneapolis, Minnesota, United States). The CHO (A), surfactant (Span 60, B), and bile salt (SDC, C) were taken as independent factors, whereas their effects were measured on VS (nm) and EE, (%), as shown in Table 4. A total of 17 runs were obtained from the software after placing the variables (Table 1). All 17 runs were prepared and analyzed for VS and EE. The values of responses were again placed in the BBD of the Design Expert to find the projected values, experimental models, polynomial equations, and 3D surface plots. The experimental models, viz. linear, 2nd order (2F1), and quadratic, were investigated to select the best-fitting model.

### 4.4. Characterization of CIP-BLO

VS, PDI, and zeta potential of BLO were analyzed by the zeta-sizer (HAS 3000, Malvern Instruments, Malvern, UK). The diluted sample (20th times) was filled into a cuvette, and the VS and PDI, and zeta potential at 25 °C were analyzed. The refractive index of the medium was 1.33, and the incidence angle for the DLS measurement was 90°. The morphology of the optimized CIP-BLO was assessed by TEM (Philips CM 10, Holland). The specimen was made by a dropping of BLO dispersion on a copper grid, staining with a phosphotungstic acid solution (1.5% *w*/*v*, one drop), and eventually drying at 25 °C. Finally, the image was captured by the camera.

### 4.5. Entrapment Efficiency (%)

The EE of CIP in BLO was estimated using an indirect method based on the finding of free (un-entrapped) CIP [31]. The specific volume (10 mL) of CIP-BLO dispersion was centrifuged at 12,000 rpm for 20 min and the supernatant separated. Then, the supernatant was diluted with a mixture of methanol and chloroform (1:1), filtered (0.45 µm syringe filter, PTFE/polytetrafluoroethylene, HiMedia, Mumbai, India) and absorbance was analyzed by UV-visible spectrophotometer at 277 nm (Shimadzu, Japan model 1800). The EE is calculated by the given Equation (3).

(3) $$EE\;\left(\%\right)=\frac{Initial\;weight\;of\;CIP-Weight\;in\;supernatant\;}{Initial\;weight\;of\;CIP}\times 100$$

### 4.6. Thermal Analysis

A thermogram of CIP, CHO, SDC, and optimized CIP-BLO was obtained by a DSC machine (Mettler Toledo, OH, USA). The samples were scanned between 25–350 °C at a 10 °C/min scanning rate in the nitrogen gas inert environment. Each thermogram was recorded using the STAR-SW 12.10 application [44].

### 4.7. X-ray Diffraction Study

An X-ray diffractometer (Ultima IV diffractometer, Rigaku Inc., Tokyo, Japan) was employed for analysis of the diffractogram of CIP, CHO, SDC, and optimized CIP-BLO. Each sample was put into the instrument after being filled into a sample holder in a thin layer separately. The samples were scanned between 5–60° at the 2-theta level. The instrument was operated at 40 mA and 40 kV.

### 4.8. Storage Stability Study

The stability of the optimized CIP-BLO was tested for 6 months according to (ICH) guidelines. The BLO was filled in a vial and stored in a humidity chamber 4 ± 0.5 °C and 25 ± 2 °C/60 ± 5% RH. The BLO was taken out at definite time intervals and tested for vesicle size, EE, zeta potential, and percent drug release.

### 4.9. Preparation of CIP-BLO In-Situ Gel

The optimized CIP-BLO (CIP-BLO-opt) was converted into the sol-gel form by employing Carbopol-934P (pH-sensitive) and HPMC K100 M (viscosity enhancer) polymers in different ratios, as shown in Table 5. The pH-triggered in-situ gelation method was used in the development of the formulation [24]. As per this method, the 0.9% *w*/*v* NaCl solution was prepared, and the desired quantity of HPMC (0.2–1% *w*/*v*) was added under continuous stirring for the formation of an HPMC dispersion without any lumps. The desired quantity of Carbopol (1–1.8% *w*/*v*) was sprinkled over it and left overnight to ensure complete hydration and proper swelling. A sufficient quantity of CIP-BLO-opt dispersion (equivalent to 0.3 *w*/*v* of CIP) was incorporated into the polymeric solution. Finally, a preservative (0.02% *v*/*v* benzalkonium chloride) was added to form the final formulation.

### 4.10. Characterization of CIP-BLO In-Situ Gel

#### Gelling Strength

Gelling capacity is defined as the time required to produce the gel (change in viscosity) on contact with simulated tear fluid [STF, 2 g NaHCO_3_ 6.7 g NaCl, and 0.8 g CaCl_2_•2H_2_O in 1000 mL of water] under ocular physiological conditions [45]. The gelling strength of the developed CIP-BLO-opt-IG formulations was measured by adding 100 µL of the formulations into 2 mL of STF (pH−7.4) in a glass vial and observing visually. The time for gel formation was recorded. The production of tear fluid is a continuous process and, under normal physiological conditions, its volume is 7 µL and is replaced within 5 min with new tear fluid. It may extend to up to 400 µL/min if irritation persists in the eyes as a result of unhealthy or disease conditions [1]. In other words, the production and replacement of eye fluid is a continuous process. In such a situation, the formulation may come into contact with a greater volume of tear fluid as compared to the normal (7 µL). To simulate the physiological condition of the eyes, usually, a higher volume of STF is taken to dilute one or two drops (30–100 µL) of gel formulation. Furthermore, in the previously reported findings, researchers took approximately 30–100 µL of the formulation and diluted it up to 2 mL with STF for the examination of gelling strength [5,31,46].

### 4.11. Measurement of Clarity, pH, and Transmission

The clarity of the CIP-BLO-opt-IG formulations was evaluated by visual observation under a black-and-white background. The pH of the formulation was analyzed by A pH meter (Hanna, Europe). The % transmission of CIP-BLO-IG formulations was assessed by a UV spectrophotometer at 480 nm using STF as a blank [47].

### 4.12. Measurement of Viscosity

The Brookfield viscometer (Model LVDV–II + PRO, USA) was utilized for the analysis of the viscosity of the CIP-BLO-opt-IG formulations. The viscosity was measured in sol form at pH 5.4 ± 0.02, (25 °C) and for the gel state, the CIP-BLO-IG formulations were mixed individually with STF (1:1, 7.4 ± 0.02 37 ± 0.5 °C) [24]. The operation was performed using 61 spindle numbers [24].

### 4.13. Determination of Drug Content

A suitable quantity (1 mL) of CIP-BLO-opt-IG formulations was dissolved separately in distilled water and then centrifuged at 6000 rpm for 20 min. Then, the collected supernatant was filtered and absorbance was analyzed by UV-spectrophotometer (Shimadzu UV-1800, Japan) at 277 nm, and the drug content was calculated.

Based on the above-said parameters, the optimized in-situ gel formulation was selected and used for further evaluation.

### 4.14. In-Vitro Dissolution Study

A dialysis membrane was used for the dissolution of the optimized CIP-BLO-opt-IG, CIP-IG, and pure CIP. Before the experiment, the dialysis membrane was activated by dipping it in distilled water overnight and then fitted at one end of the test tube. The STF (100 mL) was taken in a beaker and maintained at 37 ± 0.5 °C. 1 mL of optimized CIP-BLO-opt-IG (CIP, CIP-IG, and pure CIP at 0.3% CIP *w*/*v* was filled to the test tube assembly separately, dipped in release media (just the dialysis membrane) and then rotated at 50 rpm using a magnetic stirrer. At a predetermined interval, 2 mL of the aliquot was collected and replaced with freshly prepared media in a beaker. The aliquot was filtered through a membrane filter (0.45 µm syringe filter, made up of PTFE/polytetrafluoroethylene, HiMedia, Mumbai, India) and absorbance was measured by a UV- spectrophotometer at 277 nm. Finally, the percent drug release was calculated. The release profile of the optimized CIP-BLO-opt-IG was subjected to different kinetic models (Table S2) to evaluate the release kinetics and mechanism.

### 4.15. Ex-Vivo Corneal Permeation Study

The fresh eyes of the goat (male goat, 1.5 years old, 30 kg weight) were procured from a slaughterhouse and the cornea was carefully isolated and cleaned with saline, and stored in fresh STF (pH 7.4). There are several methods that have been reported for evaluating the cornea integrity, i.e., electron transmission microscopy, iontophoresis, etc., and are performed immediately after the sacrificed animal, transportation period, and during diffusion experiments in 6 h [48]. However, in the present work, the histological study was performed after treatment with formulation and control (0.9% *w*/*v* NaCl) to check the effect on the integrity of the corneal membrane. The study was carried out using the vertical jacketed glass diffusion cell. The diffusion cell consisted of a donor (3 mL capacity) and receptor (10 mL capacity) compartment. The receptor compartment was covered by a water jacket with two openings for the inlet (lower opening) and outlet (upper opening) of water. The receptor compartment consisted of an opening (5 mm internal diameter) on its vertical surface for sampling. The STF was degassed by a bath sonicator (Aarkey Labtronix India) for 5 min and filled (10 mL) into the acceptor compartment of each diffusion cell. An excised goat cornea (0.66 cm^2^ area) was fitted between the donor and receptor compartments, clamped with a stainless-steel clamp for proper fixation, and sealed with para-film to prevent any loss. The 37 ± 0.5 °C and 50 rpm speeds were marinated throughout the experiment. The star head magnet was used for stirring the receptor solution using a magnetic stirrer. 1 mL of optimized CIP-BLO-opt-IG, CIP, CIP-IG, and pure CIP (0.3% CIP *w*/*v*) was filled into the donor compartment of the respective diffusion cell. The 1 mL of the sample was taken from the acceptor compartment at fixed time intervals (0.5, 1,2,3,4, and 6 h), and the same volume of fresh STF was added concurrently [24]. The concentration of CIP was examined by the HPLC method [49,50]. The acetonitrile and phoasete buffer (pH 2.4) (77:33 *v*/*v*) at 1 mL/min was used as the mobile phase. The Jasco HPLC system was used. It has a Jasco manual sampler coupled with a Jasco UV 2075 plus Absorbance UV detector, a Jasco PU-2080 plus controller pump and C-18 Cosmosil packed column (250 mm x 4.6 mm x 5.0 μm). Jasco Borwin version (1.5, LC-Net II/ADC system) software was used for analysis. The 20 µL of injection volume and operation was performed at 25 °C with a UV detector at 285 nm. Before using the mobile phase, it was degassed by a bath sonicator (Aarkey Labtronix India) for 5 min. The method was found to be linear over the concentration range of 1–20 µg/mL for the peak area. The intraday and interday precision had <2% RSD, confirming the sample’s acceptable stability and method consistency. The percentage recovery was 98.15–100.42. The limit of detection (LOD) and limit of quantitation (LOQ) were 0.045 and 0.135 µg/mL, respectively, demonstrating that the method can be detected and quantified even in very low drug concentrations. The % drug permeation was determined. The flux (Jss, µg/cm^2^. h), permeability coefficient (PC, cm/h), and enhancement ratio (ER) were calculated by using equations 4, 5, and 6, respectively [51,52].

(4) $$\mathrm{Flux}\;\left(\mathrm{Jss}\right)=\frac{\mathrm{Amount}\;\mathrm{of}\;\mathrm{CIP}\;\mathrm{permeated}\;\left(µg\right)\;}{\mathrm{Time}\;\left(h\right)\times \;\mathrm{area}\;\mathrm{of}\;\mathrm{corneal}\;\mathrm{membrane}}$$

(5) $$PC\;=\frac{Flux\;}{Initial\;amount\;\;of\;CIP\;}$$

(6) $$ER\;=\frac{Flux\;of\;CIP-BLO-opt-IG3\;}{Flux\;of\;comparator\;\left(CIP-IG\;or\;CIPdis\right)}$$

### 4.16. Bio-Adhesive Study

The bio-adhesion potential of optimized CIP-BLO-opt-IG was measured by the physical balance method using excised goat cornea. The cornea was fastened on the opposite side of the pan and sited for 5 min in a sample container having optimized CIP-BLO-opt-IG. The weight was constantly applied in the second pan until the cornea was separated from the optimized CIP-BLO-opt-IG. The bio-adhesiveness (dyne/cm^2^) of the optimized CIP-BLO-opt-IG was assessed using the following formula (Equation (7))

(7) $$\mathrm{Bioadhesiveness}=\frac{Weight\;\left(g\right)\;required\;to\;detached\;cornea\;\times \;gravity\;}{Corneal\;surface\;area}$$

### 4.17. Corneal Hydration Level (CHL) Study

The capability of the cornea to tolerate the developed formulation was assessed by a corneal hydration study. The gravimetric method was used using excised goat cornea [41]. The goat cornea was immersed in optimized CIP-BLO-opt-IG for 2 h, then removed and weighed (wet weight). After that, cornea was kept at 90 °C for drying overnight and weighed again (dry weight). The CHL, %) was calculated by the given Equation (8):

(8) $$\%\;CHL=1-\left(\frac{Dry\;weight}{Wet\;weight}\right)\times 100\;$$

### 4.18. Histology Study

The histology of the treated excised goat cornea was done to evaluate any internal corneal membrane injury. The fresh goat cornea was incubated with optimized CIP-BLO-opt-IG and NaCl (0.9% *w*/*v*, control) for 6 h. After that, they were rinsed with STF and immersed in an 8% *v*/*v* formalin solution. The cornea was dehydrated with alcohol and embedded in paraffin wax to form a solid block. Then, the cornea was stained with hematoxylin and eosin. The image was captured using a Motic digital microscope at 10× magnification (MOTIC, DMB3, Pal system, Japan).

### 4.19. Ex-Vivo Ocular Tolerance

The Hen’s egg test chorioallantoic membrane (HET-CAM) test was applied to determine the ocular toxicity of the formulation. It is an alternative to the Draize test. To perform this test, fertilized eggs were used and obtained from a local poultry farm. The egg was incubated at 37 ± 0.5 °C and 55 ± 5% RH for 10 days with daily rotation and removed from the incubator on the tenth day. The eggshell was detached from the air chamber side. The 0.9% NaCl solution was dropped over the membrane and withdrawn without harming the CAM. The CAM was divided into three sections. The first section was treated with 2–3 drops of 0.9% *w*/*v* NaCl (negative control), the second with 0.1M NaOH (positive control), and the third with optimized CIP-BLO-opt-IG. Each section was visually examined for any changes (hemorrhage, vasoconstriction, and coagulation) in CAM lasting up to 5 min. The score was assigned as follows: no hemorrhage (score 0–0.9) indicates non-irritant, visible membrane discoloration (score 1–4.9) indicates slightly irritant; partial hemorrhage (score 5–8.9) indicates moderately irritant, and complete hemorrhage (score 9–21) indicates extremely irritant [53].

### 4.20. Biological Compatibility Evaluation

The biological compatibility of CIP-BLO-opt-IG3 was examined using goat blood. A smear was prepared by mixing a drop of optimized CIP-BLO-opt-IG and 0.9% *w*/*v* NaCl (control) with a drop of blood on a clean glass slide and stained with Leishman’s stain and stood for 5 min. The excess dye was soaked with tissue paper and air-dried. The red blood cells were examined under a light microscope at 40X magnification for any damage.

### 4.21. Sterility Study

The sterility test of the formulation was performed using fluid thioglycolate (for aerobic bacteria) and soybean casein digest media (for anaerobic bacteria and fungi). Each medium was prepared and sterilized in an autoclave (Astell, England, 121 °C for 15 min). Under aseptic conditions, the optimized CIP-BLO-opt-IG (0.5 mL) was inoculated into each medium (10 mL in each test tube) and incubated at 30–35 °C for fluid thioglycollate medium and at 20–25 °C for soybean casein digest medium for 14 days. After 14 days, each test tube was assessed visually for the presence of any growth.

### 4.22. Antimicrobial Evaluation

A comparative antimicrobial study of optimized CIP-BLO-opt-IG and pure CIP) was performed against *Pseudomonas aeruginosa* (*P. aeruginosa*, gram-negative) and *Staphylococcus aureus* (*S. aureus*, Gram-positive) using the cup plate method. Nutrient agar medium was sterilized in an autoclave (Astell, England) at 121 °C for 15 min. The 10 mL of medium was filled aseptically into Petri plates. The micro-organisms (*P. aeruginosa and S. aureus*) were inoculated into each plate, mixed and allowed to solidify. After solidification, the two cups (4 mm, one for optimized CIP-BLO-opt-IG and one for pure CIP dispersion) were made on each plate by using a sterile borer. The optimized CIP-BLO-opt-IG (0.3% *w*/*v* CIP) and pure CIP (0.3% *w*/*v* CIP) were filled into respective cups and allowed to stand for 2 h. Then, they were incubated at 37 °C for 24 h in an incubator (Binder, Camarillo, CA, USA). Finally, after 12 and 24 h, the zone of inhibition was measured.

### 4.23. Statistical Evaluation

Statistical evaluation was carried out using GraphPad software (version 5, “Graph Pad, San Diego, CA”, USA). All the data w expressed as mean ± SD. The *p* < 0.05 considered for significant. One-way ANOVA with the student’s t-test was applied for comparison between two formulations/groups.

## Figures and Tables

**Table 1:** Showing the composition of CIP-BLO formulations with their experimental and predicted values of responses.

Batch Code	Independent Variables			Independent Variables			
	CHO (mg)	Span-60 (mg)	SDC (mg)	VS (nm)		EE (%)	
				Exp. Values	Pred. Values	Exp. Values	Pred. Values
CIP-BLO1	10.00	40.00	20.00	148.3 ± 12.5	149.51	67.1 ± 2.7	66.71
CIP-BLO2	30.00	40.00	20.00	259.6 ± 18.1	260.05	85.3 ± 2.1	85.30
CIP-BLO3	10.00	60.00	20.00	113.3 ± 7.9	112.94	76.2 ± 3.1	76.11
CIP-BLO4	30.00	60.00	20.00	191.7 ± 14.7	190.55	88.1 ± 1.9	89.41
CIP-BLO5	10.00	50.00	15.00	149.6 ± 9.3	149.06	68.1 ± 1.4	68.40
CIP-BLO6	30.00	50.00	15.00	239.5 ± 16.8	240.10	85.0 ± 2.7	85.69
CIP-BLO7	10.00	50.00	25.00	120.8 ± 6.2	126.64	70.3 ± 2.3	70.41
CIP-BLO8	30.00	50.00	25.00	223.1 ± 14.8	223.76	86.3 ± 2.8	85.01
CIP-BLO9	20.00	40.00	15.00	233.0 ± 19.3	232.48	75.7 ± 1.8	75.75
CIP-BLO10	20.00	60.00	15.00	161.0 ± 12.4	162.01	80.3 ± 3.1	80.04
CIP-BLO11	20.00	40.00	25.00	196.6 ± 8.3	195.67	73.7 ± 2.4	73.95
CIP-BLO12	20.00	60.00	25.00	159.5 ± 9.2	160.07	83.3 ± 3.1	83.17
CIP-BLO13	20.00	50.00	20.00	170.6 ± 7.6	171.04	77.2 ± 2.6	77.59
CIP-BLO14	20.00	50.00	20.00	170.0 ± 9.2	171.04	78.5 ± 2.9	77.59
CIP-BLO15	20.00	50.00	20.00	172.2 ± 7.3	171.04	77.6 ± 2.4	77.59
CIP-BLO16	20.00	50.00	20.00	170.3 ± 9.7	171.04	78.2 ± 2.3	77.59
CIP-BLO17	20.00	50.00	20.00	171.9 ± 8.3	171.04	77.5 ± 3.2	77.59

* Values are shown as average ± SD, *n* = 3, SDC = sodium deoxycholate, Exp. = Experimental, Pred. = Predicted. VS = Vesicle size, EE = Entrapment efficiency.

**Table 2:** Analysis of variance table for responses with the best-fitted quadratic model.

Source	Vesicle Size (nm)					Entrapment Efficiency (%)				
	Sum of the Square	DF	Mean Square	F-Value	*p*-Value	Sum of the Square	DF	Mean Square	F-Value	*p*-Value
Model	25,397.74	9	2821.97	1937.80	<0.0001 *	625.84	9	69.54	514.71	<0.0001 *
Residual	10.19	7	1.46	-	-	0.95	7	0.14	-	-
Lack of Fit	6.31	3	2.10	2.17	0.2347 **	0.53	3	0.18	1.73	0.2984 **
Pure Error	3.88	4	0.97	-	-	0.41	4	0.10	-	-
Total	25,407.94	16	-	-	-	626.78	16	-	-	-

* Significant *p* < 0.05, ** Nonsignificant *p* > 0.05.

**Table 3:** Various evaluation parameters of CIP-BLO-opt-IG.

Formulation Code	Clarity	% transmission	Gelling Strength		Viscosity (cP)		Drug Content (%)
			Sol (5.4 ± 0.02)	Gel (STF, pH 7.4 ± 0.02)	Sol (5.4 ± 0.02)	Gel (STF, pH 7.4 ± 0.02)	
CIP-BLO-opt-IG1	Transparent/Clear	92.82 ± 2.36	−	+	10.7 8 ± 1.27	26.43 ± 3.86	94.56 ± 3.26
CIP-BLO-opt-IG12	Transparent/Clear	94.52 ± 1.73	−	++	25.42 ± 2.42	145.85 ± 9.48	95.72 ± 2.84
CIP-BLO-opt-IG13	Transparent/Clear	98.65 ± 1.92	−	+++	55.43 ± 6.28	266.32 ± 9.78	98.92 ± 2.43
CIP-BLO-opt-IG14	Not Transparent	91.15 ± 2.81	+	++++	107.43 ± 5.81	387.73 ± 15.54	99.31 ± 1.60
CIP-BLO-opt-IG15	Turbid	81.83 ± 2.48	+	++++	128.51 ± 2.03	431.63 ± 14.23	99.11 ± 1.01

(−), no gelation remains as liquid, (+) gelation formed in one minute and dissolved after 1 h, (++) gel formed in few seconds, and dissolved in 1 h, (+++), gel form in few seconds and stable > 24 h, (++++), the gel formed rapidly and was stable for a long time > 24 h but was hard and untransparent.

**Table 4:** Ingredient, levels, and constraints for optimization of formulation using BBD.

Independent Variable			Dependent Variable	Goal
Name and Unit	Level			
	Lower (−1)	Upper (+1)		
Cholesterol (CHO, mg)	10	30	Vesicle size (VS, nm)	Optimum
Surfactant (span 60, mg)	40	60	Entrapment efficiency (EE, %)	Maximum
Bile salt (SDC, mg)	15	25	−	−

Here surfactant is Span 60, Bile salt is Sodium deoxycholate.

**Table 5:** Composition of in-situ gel.

Ingredients	Formulation Code				
	CIP-BLO-opt-IG1	CIP-BLO-opt-IG2	CIP-BLO-opt-IG3	CIP-BLO-opt-IG4	CIP-BLO-opt-IG5
Carbopol 934P (% *w*/*v*)	1.0	1.2	1.4	1.6	1.8
HPMC K100M (% *w*/*v*)	0.2	0.4	0.6	0.8	1.0
Benzalkonium Chloride (% *v*/*v*)	0.02	0.02	0.02	0.02	0.02
Sodium chloride (g)	0.90	0.90	0.90	0.90	0.90
Distilled water (mL)	100	100	100	100	100

## Supplementary Materials

The following supporting information can be downloaded at: https://www.mdpi.com/article/10.3390/gels8110687/s1. Figure S1A–F: Showing the process diagnostic plots between predicted and actual (a and d); residual and predicted (b and e), and residual and run responses (c and f) for vesicle size entrapment efficiency, Supplementary Figure S2. DSC thermogram of (A) ciprofloxacin, (B) cholesterol, (C) sodium deoxycholate, and (D) ciprofloxacin-loaded bilosomes. The ciprofloxacin-loaded bilosomes thermogram reveals that the CIP encapsulated in bilosomes matrix, Table S1. Statistical model summary of selected dependent variables. Table S2. Kinetics model applied on the in-vitro drug release profile of optimized CIP-BLO-opt-IG3 formulation. Table S3. Showing the THE-CAM irritation score of the CIP-BLO-opt-IG3, compared to Normal Saline (0.9%) negative control, 0.1 M NaOH, and Positive control.
