# Supplementary material for: Development of Ciprofloxacin-Loaded Bilosomes In-Situ Gel for Ocular Delivery: Optimization, In-Vitro Characterization, Ex-Vivo Permeation, and Antimicrobial Study
Source: Gels. 2022 Oct 25;8(11):687. doi: 10.3390/gels8110687 (PMC9688993; doi:10.3390/gels8110687)
Supplement: Supplementary file 1 [file gels-08-00687-s001.zip › gels-1942463-supplementary.pdf]

Supplementary figures and tables

Development of Ciprofloxacin-loaded bilosomes *in-situ* gel for ocular delivery: optimization, *in-vitro* characterization, *ex-vivo* permeation, and antimicrobial study

Omar Awad Alsaidan[1], Ameeduzzafar Zafar[1], Mohd Yasir[2], Sami I. Alzarea[3], Mohammed Alqinyah[4] and Mohammad Khalid[5]

[1]Department of Pharmaceutics, College of Pharmacy, Jouf University, Sakaka 72341, Al-Jouf, Saudi Arabia

[2]Department of Pharmacy, College of Health Sciences, Arsi University, Asella 396, Ethiopia

[3]Department of Pharmacology, College of Pharmacy, Jouf University, Sakaka, 72341, Al-Jouf, Saudi Arabia

[4]Department of Pharmacology and Toxicology, College of Pharmacy, King Saud University, Riyadh, Saudi Ara-bia

[5]Department of Pharmacognosy, College of Pharmacy, Prince Sattam Bin Abdulaziz University, Al-Kharj 11942, Saudi Arabia

* Correspondence: Ameeduzzafar Zafar, Email: azafar@ju.edu.sa

Supplementary figures

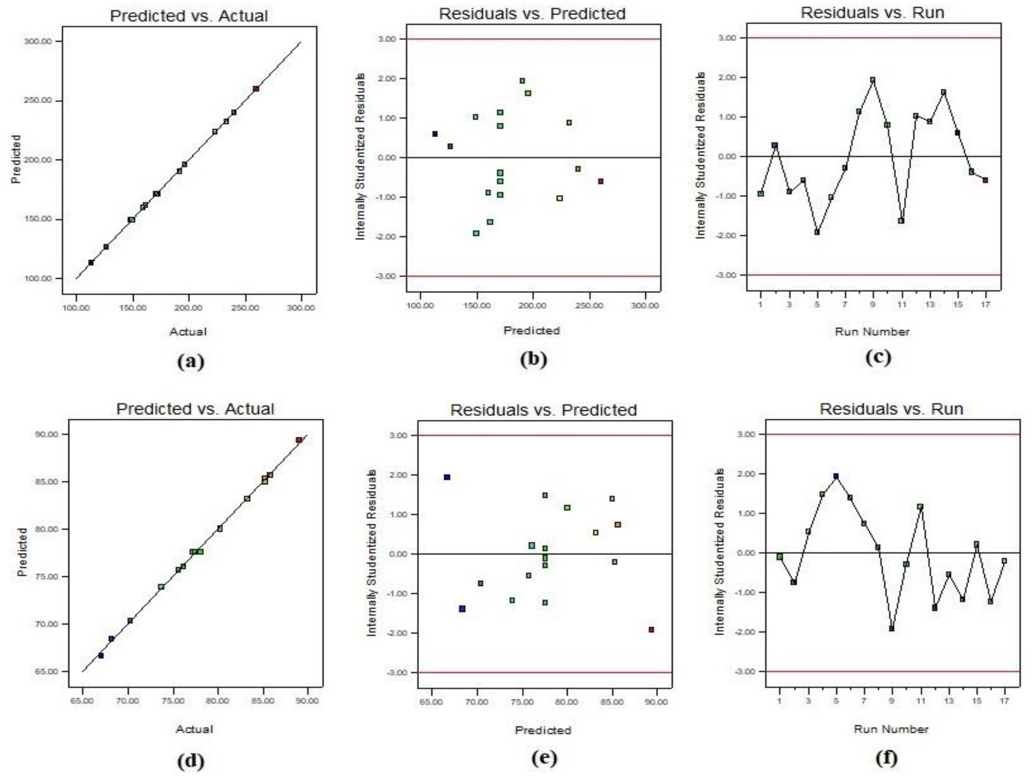

Supplementary figure S1A-F: Figure showing the process diagnostic plots between predicted and actual (a and d); residual and predicted (b and e), and residual and run responses (c and f) for vesicle size entrapment efficiency.

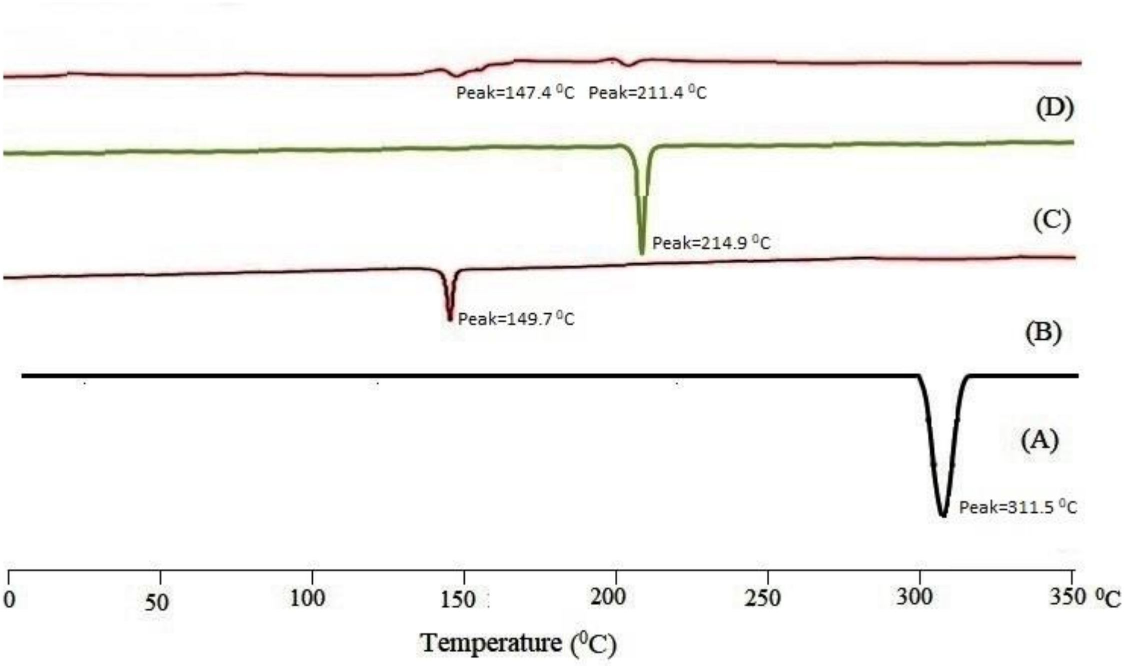

Supplementary Figure S2. DSC thermogram of (A) ciprofloxacin, (B) cholesterol, (C) sodium deoxycholate, and (D) ciprofloxacin-loaded bilosomes. The ciprofloxacin-loaded bilosomes thermogram reveals that the CIP encapsulated in bilosomes matrix

Supplementary tables

Table S1. Statistical model summary of selected dependent variables.

FP with unit	Regression parameters	Models			Best fitted model
		Linear	2FI	Quadratic	
**VS (nm)**	**SD**	10.12	8.65	1.21	Quadratic model
	R^2	0.9476	0.9706	0.9996	
	Adjusted R^2	0.9355	0.9529	0.9991	
	Predicted R^2	0.9053	0.9112	0.9958	
	% CV	-	-	0.67	
	Adeq Precision	2407.02	2257.19	107.02	
	P-value	< 0.0001	0.1097	< 0.0001	
EE (%)	SD	1.41	1.05	0.37	Quadratic model
	R^2	0.9587	0.9825	0.9985	
	Adjusted R^2	0.9492	0.9720	0.9966	
	Predicted R^2	0.9163	0.9213	0.9853	
	% CV	-	-	0.47	
	Adeq Precision	52.45	49.33	9.19	
	P-value	< 0.0001	0.0298	0.0004	

FP= Formulation parameters, VS= Vesicle size, EE= Entrapment efficiency.

Table S2. Kinetics **m**odel applied on *the in-vitro* drug release profile of optimized CIP-BLO-opt-IG3 formulation.

S.No	Model's name	Graphs	R^2	Remark
1	Zero-order	% Cum Drug Release V/S Time	0.8896	
2	First-order	% Log Cum Drug Remaining V/S Time	0.9611	
3	Higuchi model	% Cum Drug Release V/S Square root time	0.8877	
4	Korsmeyer-Peppas model	% Cum log drug release V/S log time	0.9667 n=6466	The best fitted
**5**	Hixon – Crowell model	% Cube root Drug remaining V/S Time	0.9564	

Table S3. Showing the THE-CAM irritation score of the CIP-BLO-opt-IG3, compared to Normal Saline (0.9%) negative control, 0.1M NaOH, and Positive control.

**Name of sample**	**Egg**	**Scores of the study**										**Overall score**
		**Mild irritant (a)**				**Hyperaemia (b)**			**Coagulation (c)**			
		**Time (min)**				**Time (min)**			**Time (min)**			
		**0**	**0.5**	**2**	**5**	**0.5**	**2**	**5**	**0.5**	**2**	**5**	
**CIP-BLO-opt-IG3**	**Egg 1**	0	0	0	0.4	0	0	0	0	0	0	0.4
	Egg 2	0	0	0	0.4	0	0	0	0	0	0	
	Egg 3	0	0	0	0.4	0	0	0	0	0	0	
	Mean score	0	0	0	0.4	0	0	0	0	0	0	
0.1M NaOH Positive control	Egg 1	0	0	0	4	0	0	7	0	0	6	16.93
	Egg 2	0	0	0	5	0	0	5	0	0	6	
	Egg 3	0	0		5	0	0	6	0	0	7	
	Mean score	0	0	0	4.6	0	0	6	0	0	6.33	
Normal Saline (0.9%) negative control	Egg 1	0	0	0	0.5	0	0	0	0	0	0	0.5
	Egg 2	0	0	0	0.5	0	0	0	0	0	0	
	Egg 3	0	0	0	0.5	0	0	0	0	0	0	
	Mean score	0	0	0	0.5	0	0	0	0	0	0	
